# Stress-echocardiography in idiopathic dilated cardiomyopathy: instructions for use

**DOI:** 10.1186/1476-7120-3-3

**Published:** 2005-02-10

**Authors:** Aleksandar N Neskovic, Petar Otasevic

**Affiliations:** 1 Cardiovascular Research Center, Dedinje Cardiovascular Institute, Belgrade, Serbia and Montenegro; 2 Belgrade University Medical School, Belgrade, Serbia and Montenegro

**Keywords:** stress-echocardiography, dilated cardiomyopathy, prognosis

## Abstract

A number of studies have suggested that stress-echocardiography may be used for prognostic stratification in patients with idiopathic dilated cardiomyopathy. There is no consensus on which protocol or which measurements of left ventricular contractile reserve to use. The most frequently used protocol is low-dose dobutamine stress-echocardiography, and most commonly used measures of left ventricular systolic performance are ejection fraction, wall motion score index and cardiac power output.

Stress-echocardiography has been shown to predict improvement in cardiac function in patients with recently diagnosed dilated cardiomyopathy, as well as to predict which patients will benefit from the treatment with beta-blockers. Most importantly, stress-echocardiography can identify patients with worse prognosis in terms of cardiac death and need for transplantation. Additionally, contractile reserve is closely correlated with maximal oxygen consumption and can even be used for further stratification in patients with maximal oxygen consumption between 10 and 14 ml/kg/min.

Future studies are needed for head-to-head comparison of various protocols in an attempt to make standardization in the assessment of patients with dilated cardiomyopathy.

## 

Epidemiologic data from United States indicate that idiopathic dilated cardiomyopathy (DCM) is diagnosed in approximately 36/100.000 persons each year, and that it is responsible for more than 10.000 deaths per year [1]. Faced with the fact that the number of patients with DCM is constantly increasing [2], accurate assessment of patient's current status and prognosis is of the utmost importance for the implementation of optimal therapeutic algorithm as well as for the optimal utilization of resources.

## Why stress-echocardiography?

There is a widespread belief that maximal oxygen consumption, assessed by cardiopulmonary testing, is one of most, if not the most important prognostic variables in DCM patients [3]. Maximal oxygen consumption is traditionally used for selection of patients for cardiac transplantation, with a values less than 12–14 ml/kg/min indicating poor prognosis and need for transplantation [4,5]. This approach is based upon assumption that maximal oxygen consumption during cardiopulmonary testing is determined exclusively by cardiovascular factors.

However, it appears that other factors have considerable influence on maximal oxygen consumption, since it has been shown that regular physical exercise can augment oxygen consumption with little or no impact on other parameters of cardiovascular function [6]. Additionally, a normal blood flow through lower limbs was found in patients who stopped cardiopulmonary testing because of fatigue, indicating skeletal muscle dysfunction rather than pump failure [7] Patient's age and sex, as well as muscle mass are also shown to have strong influence on performance during cardiopulmonary testing [8]. Furthermore, it appears that patients with various degrees of cardiovascular impairment may yield similar maximal oxygen consumption, suggesting that there is a role for other procedures in risk stratification of patients with DCM [9].

A number of studies have shown that assessment of ventricular contractile reserve by means of stress-echocardiography may refine prognosis in patients with left ventricular systolic dysfunction [10-12]. Nevertheless, stress-echocardiography is widely underused in routine work-up in patients with heart failure, probably because of the unfamiliarity with the technique in this clinical setting.

The aim of this review is to put stress-echocardiography in DCM patients in clinical context, to give practical tips how to perform it and what to measure, as well as to try to define its role in everyday clinical practice.

## How to perform stress-echocardiography in DCM?

Unlike protocols for stress-echocardiography for coronary artery disease, there is no consensus about the protocol to be used in patients with left ventricular systolic dysfunction.

The majority of authors have used either low- or high-dose dobutamine echocardiography [13,14]. Low-dose is usually defined as 10 mcg/kg/min of dobutamine [13], although some authors also consider 20 mcg/kg/min as a low-dose infusion [15] High-dose is uniformly defined as 40 mcg/kg/min [10]. There is no consensus either on duration of each stage of dobutamine infusion, since some authors use 3-minute stage [16] while the others use 5-minute stage [10] Most authors withdraw beta-blockers prior to stress-echocardiography, but some do not [15] Atropine is generally not used to achieve submaximal heart rate.

Exercise testing has been used for the assessment of left ventricular contractile reserve but in conjuction with radionuclide angiography [17] and hemodynamic measurements [18]. The usual protocol is performed on supine bicycle in incremental stages of 25 W lasting three minutes each. There are no data about the value of exercise stress echocardiography in DCM patients. It can be assumed that exercise stress-echocardiography would have the same limitations as cardiopulmonary testing.

Dipyridamole stress-echocardiography has been proposed for stratification of patients with DCM. Standard high-dose dipyridamole protocol (0.84 mg over 10 minutes) has been used for the assessment of contractile reserve [19].

## What to measure?

All studies on stress-echocardiography in DCM measured contractile reserve of the left ventricle. Contractile reserve is defined as the difference between values of an index of left ventricular contractility during peak stress and its baseline values. There is no consensus on what index to use.

### Ejection fraction

This is the most frequently used index of left ventricular performance. However, it may not accurately reflect left ventricular contractility since it is heavily dependent on loading conditions [20] which is particularly important in patients with heart failure for the following reasons. First of all, mitral regurgitation is frequent in these patients, and can lead to overestimation of left ventricular contractility due to rise in ejection fraction caused by changes in loading conditions (higher preload, lower afterload) [21] Secondly, activation of neuroendocrine compensatory mechanisms may increase afterload, which in turn may subsequently decrease ejection fraction [22] Thirdly, left ventricular preload is dependant upon interventricular interaction which is exaggerated in cases of pulmonary hypertension [23] a frequent finding in DCM patients. Furthermore, dobutamine has variable influence on afterload, since it has been shown that it may decrease afterload by 10% in patients with mild heart failure, but may also increases afterload by 5% in patients with severe heart failure [24].

Despite all these potential drawbacks, the change in ejection fraction during stress has been shown to have crucial prognostic significance in patients with DCM. It is generally accepted that increase in ejection fraction by ≥ 5% or change from baseline ejection fraction by ≥ 20% during stress-echocardiography identifies patients with preserved left ventricular contractile reserve and better prognosis. Ejection fraction should be assessed by Simpson biplane formula.

### Wall motion score index

Wall motion score index has been traditionally used in stress-echocardiography for the detection of coronary artery disease [25]. Only two reports used this index of left ventricular contractility to assess prognosis and functional recovery of DCM patients [10,15]. Wall motion score index was assessed in a standard manner, by using 16 segment model of the left ventricle according to the recommendations given by American Society of Echocardiography [26]. The major potential drawback for use of this index is semiquantitive assessment of wall motion, which is even more subjected to inter- and intraobserver variability in DCM patients due to preexisting wall motion abnormalities and substantial number of patients with left bundle branch. It has been suggested that dobutamine induced change in wall motion score index of ≥ 0.44 identifies patients who will do better during the follow-up.

### Cardiac power output

This index is not sensitive to changes in afterload, and after optimization for preload accurately reflects contractile properties of the myocardium [27]. Noninvasive calculation of cardiac power output is relatively complex and requires special instrumentation [11]. The most practical formula to calculate cardiac power output in Watts was suggested by Cook and coworkers [28]:

*Cardiac power output *= *(cardiac output × mean arterial pressure) × 2.22 × 10 *^-3^

where cardiac output is calculated by multiplying aortic velocity-time integral by aortic valve area, and mean arterial pressure is calculated in a standard manner.

The major problem with this index is that its calculation is time consuming, requires skilled echocardiographer, and is subjected to numerous sources of error. Furthermore, very few cardiologist are familiar with this index which precludes its wider use. Suggested cut-off point between patients with respect to prognosis is dobutamine induced change in cardiac power output of ≥ 1 W.

## Prognostic significance

There is no doubt that change in left ventricular contractility during stress has considerable prognostic significance and may have profound effect on therapeutic strategy. We will review available data according to the means how contractile response was elicited.

### Low-dose dobutamine

Most authors prefer low-dose dobutamine stress-echocardiography, probably because it is considered safe and is not time consuming. A report by Paelinck and coworkers has suggested that low-dose dobutamine can identify patients with atrial fibrillation induced dilated cardiomyopathy who will improve following restoration of sinus rhythm [29]. These authors concluded that low-dose dobutamine may be used to identify patients with tachycardiomyopathy.

**Figure 1:**
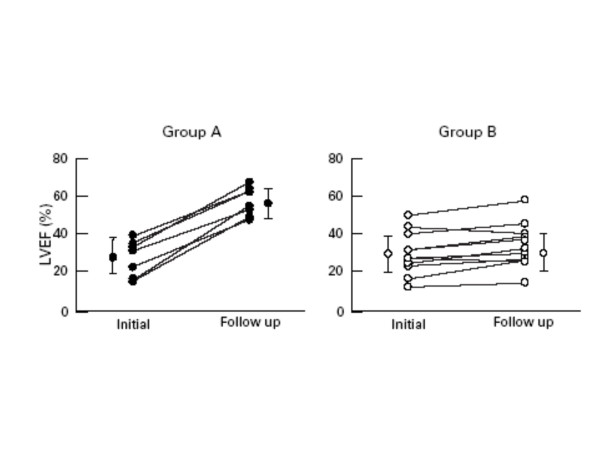
Change in ejection fraction during follow-up in patients wih preserved (group A) and diminished (group B) contractile reserve. *Abreviations: LVEF, left ventricular ejction fraction. From: Kitaoka H, Takata T, Yabe N, Hitomi N, Furuno T, Doi YL: ****Low dose dobutamine stress echocardiography predicts the improvement of left ventricular systolic function in dilated cardiomyopathy. ****Heart 1999;****81****:523-27*.

It has been shown, in a small number of patients, that changes in left ventricular wall motion score index and ejection fraction during low-dose dobutamine echocardiography are predictive of improvement of left ventricular systolic performance during medium term follow-up (Figure 1) [15]. Since the degree of beta-receptor downregulation and desenzititation is a marker of progressive deterioration of left ventricular systolic function [30], the authors hypothetized that improvement in contractility during dobutamine infusion is greater in patients with preserved beta-receptor function who will subsequently show improvement in systolic performance.

These findings are further extended, so it has been shown that the presence of myocardial contractile reserve identifies patients who will respond favorably to beta-blocker therapy [31]. Furthermore, Drozd and coworkers have demonstrated that the incidence of cardiac death or need for cardiac transplantation is lower in patients with preserved contractile reserve. In this paper, multivariate analysis identified left ventricular end-systolic volume of less than 150 ml after dobutamine infusion and no decrease of left ventricular end-diastolic volume after dobutamine infusion as significant predictors of combined end-point [32].

**Figure 2:**
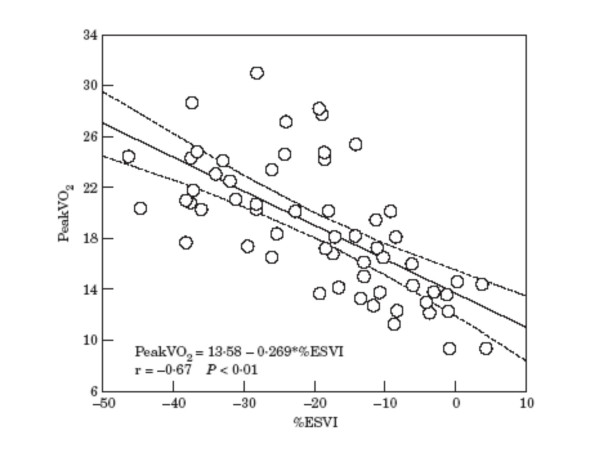
Linear correlation with 95% confidence interval between peak oxygen consumption and percent change in end-systolic volume index. *Abreviations: % ESVI, percent change in end-systolic volume index; peak VO2, peak oxygen consumption. From: Scrutinio D, Napoli V, Passantino A, Ricci A, Lagioia R, Rizzon P: ****Low-dose dobutamine responsivness in idiopathic dilated cardiomiopathy: relation to exercise capacity and clinical outcome. ****Eur Heart J 2000;****21****:927-34.*

Contractile reserve has been shown to correlate well with peak oxygen consumption (Figure 2) [16]. At multivariate analysis in this report, only percentage change in end-systolic volume index was significantly associated with occurrence of cardiac death or hospitalization for worsening heart failure. The area under receiver-operating characteristic curve was similar for percentage change in end-systolic volume index and peak oxygen consumption (0.86 ± 0.04 vs. 0.80 ± 0.06). Additionally, a report by Paraskevidis and coworkers suggested that low-dose dobutamine may further refine prognosis in patients with maximal oxygen consumption between 10 and 14 ml/kg/min [13]. This finding may be used for prioritization of patients for cardiac transplantation.

### High-dose dobutamine

Use of high-dose dobutamine is not associated with serious complications in DCM patients, and has an overall feasibility of 88.7%. The most common adverse event requiring discontinuation of dobutamine infusion is occurrence of complex ventricular arrhythmias, which was noted in 8% of patients (frequent multifocal ventricular extrasystoles in 6.4% and nonsustained ventricular tachycardia in 1.6%) [10]. Although there are no data on the association of complex ventricular arrhythmias and serum potassium concentrations, it may be postulated that complex arrhythmias are more frequent in potassium depleted patients. Therefore, it appears prudent to check serum potassium level prior to high-dose dobutamine stress-echocardiography. Hypotension, defined as decrease in systolic blood pressure by more than 30 mmHg, is very rare in the absence of complex ventricular arrhythmias and, in authors experience, occurs in less than 1% of patients with angiographically documented idiopathic DCM. Potential advantage of high-dose, as compared to low-dose, dobutamine echocardiography is that it may evoke more complete contractile response.

Very intriguing finding is that early in the course of DCM, dobutamine induced change in left ventricular contractile response and geometry is able to predict late spontaneous recovery of left ventricular systolic performance [14]. It is interesting that this study confirmed previous findings that increased left ventricular mass is associated with better outcome in DCM [33], and suggested that the presence of left ventricular hypertrophy implies the presence of myocardial contractile reserve.

**Figure 3:**
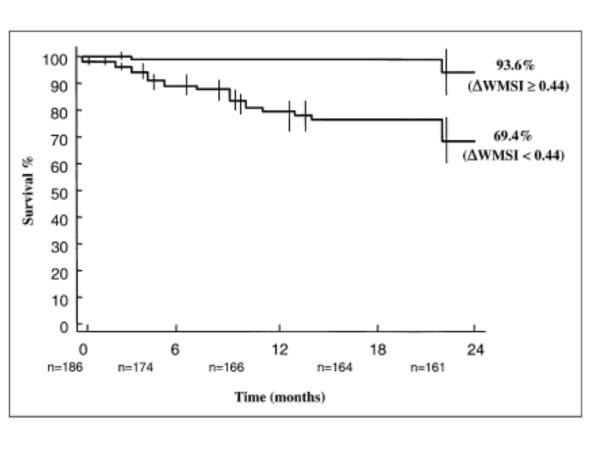
Kaplan-Meir survival curves (only cardiac deaths were considered) in patients stratified according to the dobutamine induced change in wall motion score index. *Abbreviations: ΔWMSi, change in wall motion score index. From: Pratali L, Picano E, Otašević P, Vigna C, Palinkas A, Cortigiani L, Dodi C, Bojić D, Varga A, Csanady M, Landi : ****Prognostic significance of the dobutamine echocardiography test in idiopathic dilated cardiomyopathy. ****Am J Cardiol. 200;****88****:1374-8.*

The largest study that studied prognostic significance of high-dose dobutamine included 186 DCM patients. The major findings of this study are that dobutamine induced change in wall motion score index is able to identify patients at greater risk for cardiac death during the follow-up (Figure 3), and that change in wall motion score index carries superior prognostic information than change in ejection fraction [10] Additionally, it has been reported that dobutamine induced change in ejection fraction by ≥ 8%, assessed by radionuclide ventriculography, is prognostically superior to maximal oxygen consumption in patients with severe DCM [34].

Recent data by our group demonstrate that contractile reserve indices assessed by high-dose dobutamine correlate with myocardial histomorphometric features, suggesting that contractile reserve is strongly related to the degree of hystological disruption in DCM patients. Myocyte diameter and interstitial fibrosis showed strongest correlation with change in wall motion score index (r = -0.667, p < 0.001, and r = -0.567, p = 0.004, respectively), followed by change in ejection fraction (r = -0.603, p = 0.002, and r = -0.467, p = 0.021, respectively) [35].

### Dipyridamole

It appears that dipyridamole may be used instead of dobutamine to evoke contractile response, since it is less arrythmogenic [36] better tolerated and yields similar prognostic information in patients with coronary artery disease [37]. Ability of dipyridamole to recruit contractile reserve is mediated through increase in coronary blood flow and accumulation of endogenous adenosine [38,39]. Potential advantage of dipyridamole over dobutamine stress-echocardiography is that the former is not affected by the use beta-blocking agents which are frequently used in DCM patients. Only one study recently examined ability of dipyridamole to predict prognosis in DCM patients. The authors concluded that increase in wall motion score index ≥ 0.15 during dipyridamole stress identifies patients who are more likely to survive during the mean follow-up of more than three years [19]. Reported overall feasibility of dipyridamole stress-echocardiography in this study was 99.2%, which is significantly higher than previously reported feasibility of dobutamine stress-echocardiography.

### Exercise

As previously said, there are no echocardiographic studies on exercise induced contractile response in DCM patients. However, Nagaoka and colleagues used radionuclide ventriculography to measure increase in ejection fraction during exercise in DCM patients with mild symptoms, and concluded that change in ejection fraction <4% identifies patients with worse prognosis [17]. Additionally, it has been suggested that variables, such as cardiac power output, obtained by direct hemodynamic measurements during exercise may have important prognostic implications in patients with systolic dysfunction [12].

## What about the right ventricle?

Right ventricular contribution to global cardiac performance is minor in subjects with normal or mildly depressed left ventricular systolic function, but may become more important in patients with advanced left heart failure [40]. Previous studies have suggested that right ventricular enlargement is a strong marker for adverse prognosis in DCM patients [41], as well as that right ventricular long axis excursion is predictive of exercise tolerance [42].

However, there are only limited reports of the prognostic value of right ventricular contractile reserve in patients with DCM. DiSalvo and colleagues have demonstrated that an increase in RVEF to >35% during exercise is the only independent predictor of event-free survival in patients with advanced heart failure [43]. It has been also shown that preserved right ventricular contractile reserve (measured by pressure-area relations) induced by low-dose dobutamine infusion was associated with a good 30-day outcome in patients with NYHA class IV heart failure [44].

Data from our laboratory support prognostic significance of high-dose dobutamine induced change in right ventricular fractional area change [45]. It appears that fractional area change of >9% identifies patients with more favorable outcome. More importantly, these data suggest that patients in whom contractile reserve of both ventricles is preserved will most likely have good prognosis.

## What role for stress-echocardiography?

Despite the wealth evidence that favor use of stress-echocardiography in patients with DCM, there is no clear-cut algorithm about its use in risk stratification and therapeutic strategy. The reasons for this are not clear, but probably reflect the lack of standardized protocol and measurements of left ventricular contractile reserve.

We strongly believe that stress-echocardiography should be used as a standard procedure, at least in centers which do not have access to cardiopulmonary testing, since data obtained when patients are subjected to some form of stress have far greater prognostic significance than data obtained at rest. Furthermore, stress-echocardiography should be used in patients who are not able to exercise or fail to achieve expected work load. Stress-echocardiography may also play an important role for detailed risk stratification in patients with maximal oxygen consumption of 10–14 ml/kg/min. The choice of stress-protocol, at least for the time being, should be based upon local expertise and preferences of attending physician.

## Future directions

There is an obvious lack of studies that will contribute to standardization of stress-echocardiographic protocol. Head-to-head comparison of stressors, including low- and high-dose dobutamine, dipyridamole, and exercise, has to be performed in order to rank their ability to predict prognosis. Similar comparisons have to be made for various indices of left ventricular contractility. Additionally, it is not clear should the patients be tested with or without beta-blocker therapy, and how this therapy may affect our choice of stressor. Last but not the least, novel echocardiographic techniques that can easily assess regional and global contractility, like tissue Doppler imaging and strain-rate imaging, have not yet been tested in a prospective manner.

In conclusion, stress-echocardiography can be a valuable tool for the assessment of patients with DCM, but a lot of work has to be done before it becomes a part of a routine work-up.
